# Breast cancer diagnosis as a window of opportunity for smoking cessation: analysis of changes in smoking behaviour in 736 smoking breast cancer patients

**DOI:** 10.1007/s00520-026-11030-0

**Published:** 2026-07-22

**Authors:** Raakel Lintunen, Anselm Tamminen

**Affiliations:** 1 Faculty of Medicine, Department of Clinical Medicine, University of Turku, Turku, Finland; 2 Department of Plastic and General Surgery, Turku University Hospital, Turku, Finland

**Keywords:** Breast cancer, Health behaviour, Retrospective study, Smoking cessation, Smoking, tobacco

## Abstract

**Purpose:**

Breast cancer is the most prevalent malignancy among women worldwide. Smoking increases the risk of breast cancer and is associated with poorer treatment outcomes. Although smoking behaviour and cessation have been studied among breast cancer patients, the existing evidence remains heterogeneous, and the timing of cessation around diagnosis is not fully understood. The aim of this study was to determine the proportion of breast cancer patients who quit smoking after diagnosis and to identify factors associated with smoking cessation.

**Methods:**

In this retrospective study, the smoking status of 6456 consecutive breast cancer patients from years 2010 to 2022 was evaluated. All patients were requested to complete a baseline questionnaire including information on their smoking status. The follow-up information was collected from the patient records.

**Results:**

At the time of diagnosis, 851 patients (13.2%) were actively smoking. Follow-up data were available for 736 patients (86.5%), of whom 189 (25.7%) quit smoking at the time of diagnosis despite the absence of a formal cessation program. Nearly half (44.7%) of all smoking cessation events occurring within the 5-year period before and after diagnosis took place within the year of diagnosis. Smoking cessation was significantly more likely at the time of diagnosis than during subsequent years.

**Conclusion:**

Cancer diagnosis provides a timely opportunity for smoking cessation and highlights the potential value of integrating smoking cessation interventions at the time of diagnosis.

**Supplementary Information:**

The online version contains supplementary material available at 10.1007/s00520-026-11030-0.

## Background

Breast cancer is the most common malignancy among women worldwide, with an estimated incidence of 2.3 million new cases and 664,684 deaths reported in 2022. Despite significant improvements in the prognosis of breast cancer, it remains the leading cause of cancer-related mortality among women [1].

Several risk factors for the development of breast cancer have been identified. The most significant are older age, genetic background and reproductive factors, such as early menarche, late menopause and late age at first pregnancy, as well as hormonal factors such as long-term hormone replacement therapy [2, 3]. Lifestyle choices also have an effect on the development of breast cancer. Smoking, especially when initiated at an early age, has been shown to increase the risk of breast cancer [4–6]. Additionally, it has been shown to be associated with poorer treatment outcomes [7].

The commonly used treatment modalities for breast cancer are surgery, radiation therapy, immunotherapy, hormone therapy and chemotherapy. Smoking has been found to decrease the effectiveness of all treatment strategies [8] and increase the risk of treatment-related complications and side effects [7]. Continued smoking after breast cancer diagnosis severely worsens patient prognosis [5, 9]. The likelihood of smoking cessation after cancer diagnosis varies significantly depending on the cancer type, and many patients continue to smoke after diagnosis [10, 11]. Interventions for smoking cessation are particularly important at the time of diagnosis, when the patient’s motivation to change health behaviours is at its highest [12].

Currently, interest in the impact of health behaviours on breast cancer treatment has been renewed [13, 14]. Although smoking behaviour and cessation have been studied among breast cancer patients, the existing evidence remains heterogeneous, and the timing of cessation around diagnosis is not fully understood. Understanding this timing is essential for identifying the optimal window of opportunity for promoting smoking cessation interventions.

The aim of this study was to determine the proportion of breast cancer patients who quit smoking after diagnosis, and to identify factors associated with successful smoking cessation.

## Methods

We conducted a retrospective study on smoking behaviour among breast cancer patients, diagnosed at Turku University Hospital between 2010 and 2022. A total of 6456 patients were included in the study. The baseline data of patients was received from Auria Clinical Informatics based on ICD-10 code C50 (invasive breast cancer) or D05 (carcinoma in situ). Only patients with early breast cancer receiving treatment in the surgical unit were included.

### Data collection

At the time of diagnosis and initiation of treatment, all breast cancer patients were requested to complete a baseline questionnaire regarding their smoking status, including whether they were currently smoking and, if they had quit, the timing of cessation. The most recent data on patients’ smoking status was updated manually from the hospital’s patient records between February and April in 2025.

At this point in time, patients had generally undergone regular follow-up visits with their oncologist, and information on smoking status was primarily obtained from these records. For patients without follow-up data beyond the initial breast cancer diagnosis, only the available information was included in the analysis. Only one person was responsible for collecting the data regarding smoking status to reduce variation in interpretation. In cases of interpretive uncertainty, each author assessed the situation.

Patients were categorized according to smoking status as follows: those who had never smoked regularly were classified as ‘never smoked’. Patients who had smoked but quit before diagnosis were categorized as ‘quit pre-diagnosis’. Patients who smoked daily before and after diagnosis were categorized as ‘currently smoking’. Patients who were able to temporarily quit smoking after diagnosis but later resumed and were smoking at the 1-year follow-up visit were also classified as patients who currently smoked. The patients who smoked but who had quit > 1 year post-diagnosis were categorized as ‘quit post-diagnosis’.

Those who quit within 1 year after diagnosis were categorized as ‘quit at diagnosis’. Patients who were smoking fewer than five cigarettes per day were categorized as ‘occasionally smoked’, and individuals who had only experimented with tobacco in their youth were categorized as ‘occasionally in youth’.

Additional factors collected included age at diagnosis, year of diagnosis, American Society of Anesthesiologists (ASA) classification and T- and N-staging, as well as information on whether a patient underwent breast and/or axillary surgery.

At the time of the study, breast cancer patients were not routinely referred for smoking cessation support at Turku University Hospital. In cases where a patient expressed a desire to quit smoking, referral for cessation support was possible. However, there was no standardized smoking cessation program implemented, and discussions regarding smoking during outpatient visits varied depending on individual physician preferences. Thus, we also assessed whether preoperative outpatient visits and the attending physician were associated with subsequent smoking cessation.

### Statistical analysis

Statistical analyses were performed using JMP Pro 17 analysis software. Categorical variables were compared using the chi-square test or Fisher’s exact test; continuous variables with normal distribution were compared using the *t*-test and variables not following normal distribution with Mann-Whitney *U*-test. Smoking cessation was used as the outcome variable and its association with these collected factors was analyzed using logistic regression. Patients with missing data for any given variable were excluded from analysis involving that variable, and time-dependent analyses included only patients with sufficient follow-up data. Variables with *p* < 0.15 in univariable analyses were included in the multivariable model. A backward stepwise elimination of non-significant variables was then applied until only statistically significant variables (*p* < 0.05) remained in the final model.

This study was approved by the Hospital District of Southwest Finland (T537/2022).

## Results

In total, 6456 patients were included in the study (Table 1). Median age at diagnosis was 65 years (IQR 56–74).

**Table 1:** Patient characteristics

Number of patients	6456
Age, years (median, IQR)	65 (56–74)
**Smoking status**	
Never smoked	4085 (63.3%)
Quit pre-diagnosis	1303 (20.2%)
Currently smoking	892 (13.8%)
Information missing	176 (2.7%)
**ASA Classification**	
I	1006 (15.6%)
II	3381 (52.4%)
III	1889 (29.3%)
IV	147 (2.3%)
Information missing	33 (0.5%)
**T-stage**	
DCIS	549 (8.5%)
T1a	197 (3.1%)
T1b	890 (13.8%)
T1c	2073 (32.1%)
T2	1835 (28.4%)
T3	365 (5.7%)
Information missing	547 (8.5%)
**N-stage**	
N0	3731 (57.8%)
N1	1378 (21.3%)
N2	389 (6.0%)
N3	260 (4.0%)
Nx	326 (5.0%)
Information missing	372 (5.8%)
**Breast surgery**	
Mastectomy	2082 (32.2%)
Breast conserving surgery	3537 (54.7%)
Excision of local recurrence	12 (0.2%)
Mastectomy + immediate breast reconstruction	178 (2.8%)
Bilateral	629 (9.7%)
Not performed	18 (0.3%)
**Axillary surgery**	
Axillary lymph node dissection	1875 (29.0%)
Bilateral	629 (9.7%)
Sentinel lymph node biopsy	3541 (54.8%)
Not performed	411 (6.4%)

Smoking habits before and after breast cancer diagnosis were assessed. Majority of the patients in this study had never smoked and were categorized as ‘never smoked’ [*n* = 4085 (63.3%)]. Second largest group consisted of ‘quit pre-diagnosis’ [*n* = 1048 (16.2%)]. A smaller group of patients had only smoked ‘occasionally in youth’ [*n *= 254 (3.9%)]. A small proportion of patients (42 of 6456, 0.7%) reported occasional smoking. These individuals were not included in the detailed analysis, which focused on those who smoked regularly.

In total, 851 patients (13.2%) smoked at the time of diagnosis. Patients who smoked were younger than those who did not smoke, with median age of 60 years (IQR 52–66). Post-diagnosis smoking status could not be verified from patient records for 115 (13.5%) patients, and after excluding these patients, 736 remained eligible for detailed analysis.

**Fig. 1:**
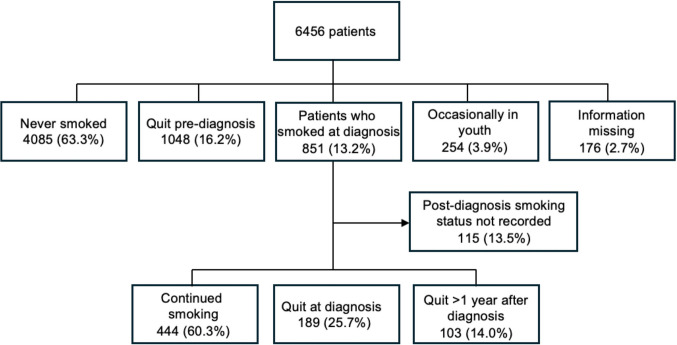
Structure of the study population

Among these patients, 292 (39.7%) quit smoking, 189 (25.7%) at diagnosis and 103 (14.0%) more than 1 year after diagnosis (Fig. 1). Medical records of patients who quit within 1 year of diagnosis were assessed and no evidence of later relapse was found. Patients were significantly more likely to quit smoking at the time of diagnosis than during the subsequent years (OR 1.49, 95% CI 1.14–1.95, *p* = 0.0038).

**Fig. 2:**
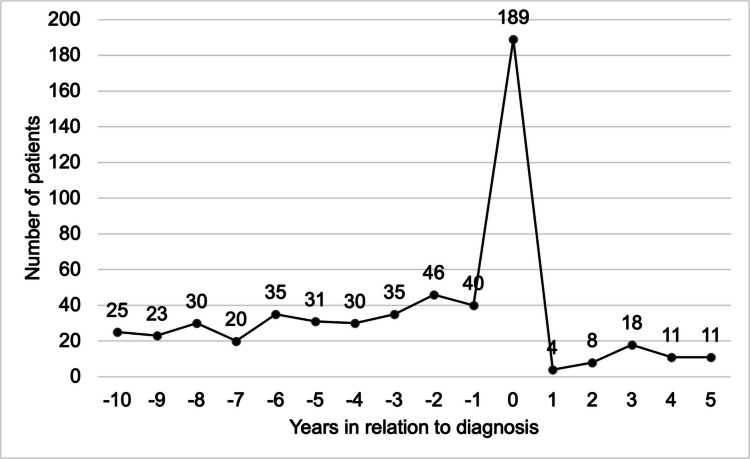
Smoking cessation by years from diagnosis

Only four patients quit smoking more than 1 but less than 2 years after diagnosis, with 8, 18, 11 and 11 patients quitting during the subsequent years, respectively (Fig. 2). Among patients who quit smoking within 5 years before to 5 years after diagnosis (*n* = 423), the year-by-year distribution of smoking cessation differed significantly from a uniform distribution (*χ*^2^ = 705.78, df = 10, *p* < 0.0001). The year of diagnosis accounted for 44.7% of all smoking cessation events within this time window (expected frequency 9.1%).

For the 444 patients who did not quit smoking, information on smoking status was verified 1 year after diagnosis for all, and for 342 patients data were available for at least 3 years after diagnosis.

Data on preoperative outpatient clinic visit in relation to smoking cessation were examined among patients who regularly smoked (*n* = 851). Patients treated by surgeons who had seen fewer than five patients, as well as patients with missing information, were excluded, leading to the exclusion of 31 patients. The analysis found no significant association between smoking cessation at diagnosis and which physician a patient had visited preoperatively (*p* = 0.137).

Statistical analyses were performed to find individual factors associated with smoking cessation. Patients with missing data were excluded from the respective statistical analyses. In univariate analyses, only younger age at diagnosis was found to be a statistically significant factor explaining smoking cessation (Table 2). Patients who had larger tumours had a tendency to quit smoking more often, but the association did not reach statistical significance (*p* = 0.0871). In multivariable logistic regression analysis including age and T-stage, only age remained independently associated with smoking cessation (Supplementary Table S1).

**Table 2:** Univariate analysis of potential risk factors

	Quit at diagnosis (*N* = 189)	Continued smoking (*N* = 547)	*p*-value
**Age, median (IQR)**	58 (50–65)	60 (52–66)	0.0227
**ASA Classification**			0.6294
ASA I	31 (16.4%)	85 (15.5%)	
ASA II	119 (63.0%)	318 (58.1%)	
ASA III	37 (19.6%)	133 (24.3%)	
ASA IV	2 (1.1%)	10 (1,8%)	
Information missing	0 (0%)	1 (0.2%)	
**T-stage**			0.0871
DCIS	7 (3.7%)	30 (5.5%)	
T1a	4 (2.1%)	16 (2.9%)	
T1b	25 (13.2%)	103 (18.8%)	
T1c	57 (30.2%)	198 (36.2%)	
T2	64 (33.9%)	137 (25.1%)	
T3	9 (4.8%)	20 (3.7%)	
Information missing	23 (12.2%)	43 (0.8%)	
**N-stage**			0.3356
N0	112 (61.2%)	330 (60.3%)	
N1	51 (27.9%)	124 (22.7%)	
N2	6 (3.3%)	33 (6.0%)	
N3	8 (4.4%)	16 (2.9%)	
Nx			
Information missing	12 (6.3%)	44 (8.0%)	
**Breast surgery**			0.1731
Mastectomy	55 (29.1%)	166 (30.4%)	
Breast conserving surgery	109 (57.7%)	335 (61.2%)	
Mastectomy + immediate breast reconstruction	5 (2.7%)	4 (0.7%)	
Bilateral	19 (10.1%)	39 (7.1%)	
Not performed	1 (0.5%)	3 (0.6%)	
**Axillary surgery**			0.3603
Axillary lymph node dissection	54 (28.6%)	168 (30.7%)	
Bilateral	19 (10.1%)	39 (7.1%)	
Sentinel lymph node biopsy	112 (59.3%)	318 (58.1%)	
Not performed	4 (2.1%)	28 (4.2%)	

Patients who quit smoking were approximately 2 years younger than those who continued smoking; the median age of patients was 58 years (IQR 50–65) for quitters and 60 years (IQR 52–66) for continuers.

**Fig. 3:**
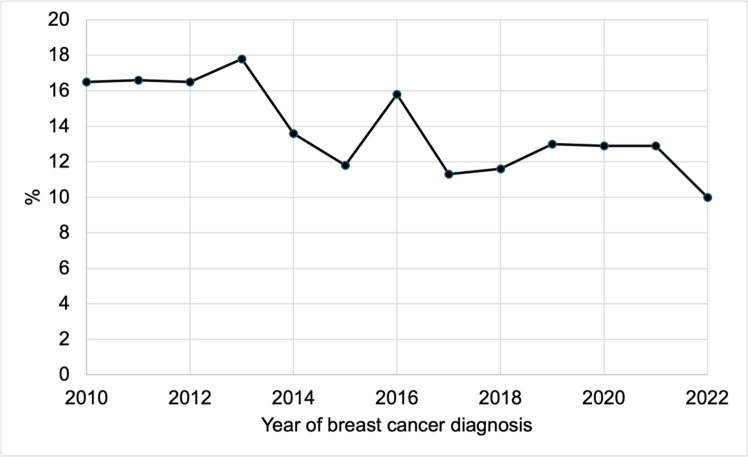
Proportion of patients who smoked by year

The proportion of patients who smoked was analyzed by year of diagnosis. A significant decreasing trend was observed over the study period (*p* = 0.0016), with the proportion decreasing from 16.5% in 2010 to 10.0% in 2022 (Fig. 3).

**Fig. 4:**
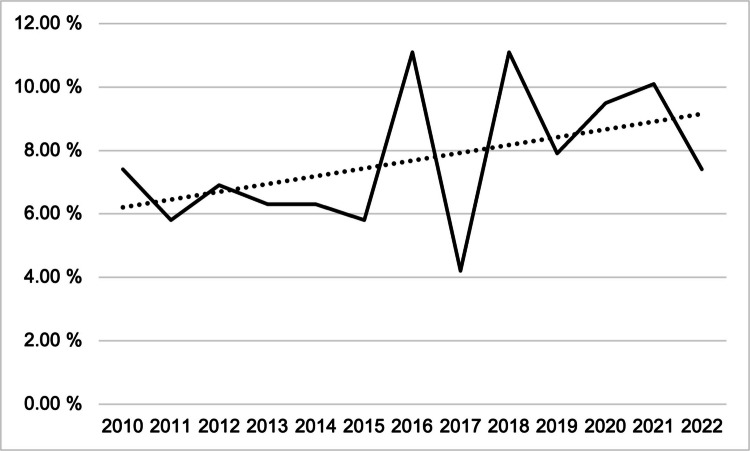
Cessation proportions throughout the 13-year study period

To examine possible change in cessation throughout the study period, year of diagnosis was analyzed. The proportion of patients who had quit smoking remained unchanged at 7.4% in both 2010 and 2022, indicating no overall change in cessation prevalence between the start and end of the study. However, considerable annual variation was observed, especially in the second half of the study period (2016–2022), with higher quit rates and greater variability (Fig. 4). Overall, smoking cessation rates fluctuated over time with no clear consistent trend (*p* = 0.1311).

## Discussion

In this large and detailed retrospective study, we found that 189, a little over a quarter of patients who smoked (25.7%, 189/736), quit smoking at the time of breast cancer diagnosis, despite the absence of a specific intervention program to support smoking cessation. Nearly half (44.7%) of all smoking cessation events within the 5-year period before and after diagnosis occurred within the year of diagnosis. Patients were significantly more likely to quit smoking at diagnosis than afterwards.

It is known that smoking cessation does not occur randomly but is strongly patterned around identifiable ‘teachable moments’, such as acute illness, hospitalization or major life transitions [15, 16]. A substantial body of evidence indicates that these moments are not only associated with increased spontaneous quitting, but also represent periods during which cessation interventions are particularly effective [15, 17]. This is also in line with the study by Cencelj Arnez et al. (2023), which reported that a substantial proportion of patients quit smoking within the first year after breast cancer diagnosis, and that targeted rehabilitation programs may further enhance cessation [18]. This suggests that heightened risk perception and motivation during these critical periods create a transient window of receptivity that can be leveraged to produce durable behaviour change. However, this opportunity is frequently missed in routine care, with many patients not receiving systematic cessation support even in clearly high-risk contexts [17, 19].

Taken together, the evidence supports the interpretation that timing is not merely a contextual factor, but a central determinant of intervention effectiveness. Therefore, integrating systematically timed, multi-component cessation interventions into clinical pathways—particularly at critical time points such moment such as breast cancer diagnosis—should be considered a priority. The present study clearly demonstrates that a breast cancer diagnosis represents precisely such a window of opportunity. Given that smoking has been shown to adversely affect breast cancer prognosis, treatment efficacy and the risk of complications, smoking cessation interventions should be systematically prioritized and actively implemented at the time of diagnosis [7–11].

Our findings further support this approach, as smoking cessation rates differed significantly before and after cancer diagnosis, remaining consistently lower throughout the 5-year observation period after diagnosis. This suggests that if a patient did not quit smoking at diagnosis, they were considerably less likely to do so thereafter. These findings highlight the importance of encouraging smoking cessation at diagnosis, as it may represent an important opportunity for behavioural change.

Previously, Tseng et al. (2012) reported somewhat higher smoking cessation rates than those observed in our study; however, only 14.1% of their study population were breast cancer patients. They also suggested that, on average, it would take 8.8 years to quit smoking after a cancer diagnosis [20]. Our study does not provide direct support for this finding, although only a small proportion of our patients had follow-up extending this far. Higher cessation rates have been reported in other studies, although they have not specifically considered breast cancer patients and some have been conducted in settings where structured smoking cessation programs were implemented [9, 21].

These interventions have been shown to be effective, especially when counselling and pharmacotherapy are combined [22, 23]. Studies have shown that patients with smoking-related cancers are more likely to quit smoking [10, 11]. The association between smoking and breast cancer has only relatively recently been established, which may explain why smoking cessation interventions were not necessarily considered a central aspect of patient management, particularly during the early years of the study period [24].

In our study, patients who smoked were younger than those who did not smoke, consistent with previous literature indicating that individuals who smoke tend to develop cancer at a younger age [25, 26]. Younger age was also significantly associated with smoking cessation at the time of diagnosis, as patients who quit smoking were younger than those who continued to smoke. However, differences in median age and IQRs between the groups were small and should therefore be interpreted with caution. Older patients in this study were more likely to have missing information regarding smoking status. While Gummerson et al. (2022) did not observe a connection between age and smoking cessation in a large US cohort [21], more recent studies suggest that smoking cessation behaviour may vary by age [20, 27, 28].

No significant associations were observed between smoking cessation and other factors, including T- and N-stage, ASA classification or the type of axillary or breast surgery. This may suggest that the cancer characteristics have little influence on the individual’s decision to quit smoking, possibly because patients may not fully understand their relevance to prognosis [29, 30].

Although surgical outcomes were not evaluated in this study, smoking is known to be closely connected to breast cancer treatment in several ways. Smoking can limit reconstructive surgical options and increase the risk of postoperative wound complications [31, 32]. A recent study by Hendizadeh et al. (2026) suggested that patients undergoing breast reconstruction may be more likely to quit smoking [33]. Together, these findings support the importance of smoking cessation support at the time of breast cancer diagnosis. Future studies could provide further insight into how smoking-related treatment risks should be considered in surgical planning.

Instead, individual characteristics such as socioeconomic status and psychosocial factors, which were not explored in this study, could have greater importance. Previous studies have shown these characteristics to be associated with smoking cessation in cancer patients [18, 34, 35]. In the present study, socioeconomic and psychosocial factors were not routinely collected from patients and were therefore unavailable for analysis. In the Finnish healthcare system, breast cancer treatment is centralized within the public sector and to a single hospital within each hospital district, which reduces selection related to access to care. However, socioeconomic factors may still influence outcomes. Turku University Hospital has been the only major hospital in the hospital district to provide breast cancer care.

### Limitations

The retrospective design of this study has limitations, as smoking data may be incomplete and was based on self-report, making it susceptible to recall bias. Furthermore, potential confounding factors, such as socioeconomic and psychosocial factors, were not accounted for. This is an important limitation as socioeconomic factors may influence smoking behaviour and motivation for cessation.

In some cases, it is possible that a patient’s smoking status was assumed to have remained unchanged, and therefore outdated information may have been recorded in the medical records, even though the patient’s smoking status may in fact have changed. In addition, even though no evidence of smoking relapse was found in the medical records of patients who quit smoking, relapse may have occurred and not recorded. This increases the possibility of error depending on how and if the question on smoking was asked, how it was recorded in the patient files, and how it was later interpreted when the information was collected for this study. Despite efforts to minimize interpretation bias, some degree of classification errors cannot be excluded. Furthermore, the relatively large data size increases the reliability of the results, even if individual errors in the data were present. To confirm the findings of this study and to assess the magnitude of the effect of cessation support, further research in a prospective setting is warranted.

Strengths of this study include the large patient population and the systematically documented data on patients’ smoking status at the time of breast cancer diagnosis. Additionally, the follow-up information on smoking status was available for more than 80% of patients, which can be considered an excellent figure given the nature of the study.

## Conclusion

About one quarter of breast cancer patients who smoked were able to quit following diagnosis despite the absence of a formal cessation program. This suggests that cancer diagnosis provides a timely opportunity for smoking cessation and highlights the potential value of integrating smoking cessation interventions at the time of diagnosis.

## Supplementary Information

Below is the link to the electronic supplementary material.

ESM 1 (DOCX  17.1 KB)

## Data Availability

The data that support the findings of this study are available on request from the corresponding author. The data are not publicly available due to privacy or ethical restrictions.
