# Supplementary material for: Breast cancer diagnosis as a window of opportunity for smoking cessation: analysis of changes in smoking behaviour in 736 smoking breast cancer patients
Source: Support Care Cancer. 2026 Jul 22;34(8):790. doi: 10.1007/s00520-026-11030-0 (PMC13391802; doi:10.1007/s00520-026-11030-0)
Supplement: Supplementary file 1 — (DOCX 17.1 KB) [file 520_2026_11030_MOESM1_ESM.docx]

**Breast Cancer Diagnosis as a Window of Opportunity for Smoking Cessation: Analysis of Changes in Smoking Behaviour in 736 Smoking Breast Cancer Patients**

**Raakel Lintunen1, Anselm Tamminen1,2**

**1 Faculty of Medicine, Department of Clinical Medicine, University of Turku, Turku, Finland**

**2 Department of Plastic and General Surgery, Turku University Hospital, Turku, Finland**

**Corresponding author: Raakel Lintunen, rwlint@utu.fi, Turku, Finland, ORCID: 0009-0007-7207-434X**

**Anselm Tamminen,** [**anselm.tamminen@utu.fi**](mailto:anselm.tamminen@utu.fi)**, Turku, Finland, ORCID: 0000-0002-3559-8407**

**Journal: Supportive Care in Cancer**

| **Variable** | **Univariable OR (95% CI)** | **p-value** | **Multivariable OR (95% CI)** | **p-value** |
| --- | --- | --- | --- | --- |
| Age | 0.98 (0.96-1.00) | 0.021 | 0.98 (0.96-1.00) | 0.039 |
| T-stage | - | 0.087 | - | 0.133 |

Supplementary Table S1. Univariable and multivariable logistic regression analyses of factors associated with smoking cessation at diagnosis
